# Sex-specific Concordance of Striatal Transcriptional Signatures of Opioid Addiction in Human and Rodent Brains

**DOI:** 10.21203/rs.3.rs-5006061/v1

**Published:** 2024-09-24

**Authors:** Ryan Logan, Micah Shelton, Nicole Horan, Xiangning Xue, Lisa Maturin, Darrell Eacret, Julie Michaud, Navsharan Singh, Benjamin Williams, Mackenzie Gamble, Joseph Seggio, Madeline Kuppe-Fish, BaDoi Phan, George Tseng, Julie Blendy, Leah Solberg Woods, Abraham Palmer, Olivier George, Marianne Seney

**Affiliations:** University of Massachusetts Chan Medical School; University of Pittsburgh; University of Pittsburgh; University of Pittsburgh; University of Pennsylvania; Wake Forest University; University of California, San Diego; University of Pittsburgh

## Abstract

Opioid use disorder (OUD) has emerged as a severe, ongoing public health emergency. Current, frontline addiction treatment strategies fail to produce lasting abstinence in most users. This underscores the lasting effects of chronic opioid exposure and emphasizes the need to understand the molecular mechanisms of drug seeking and taking, but also how those alterations persist through acute and protracted withdrawal. Here, we used RNA sequencing in post-mortem human tissue from males (n=10) and females (n=10) with OUD and age and sex-matched comparison subjects. We compared molecular alterations in the nucleus accumbens (NAc) and dorsolateral prefrontal cortex (DLPFC) between humans with OUD and rodent models across distinct stages of opioid use and withdrawal (acute and prolonged) using differential gene expression and network-based approaches. We found that the molecular signature in the NAc of females with OUD mirrored effects seen in the NAc of female mice at all stages of exposure. Conversely, males with OUD showed strong overlap in expression profile with rats in acute withdrawal. Co-expression networks involved in post-transcriptional modification of RNA and epigenetic modification of chromatin state. This study provides fundamental insight into the converging molecular pathways altered by opioids across species. Further, this work helps to disentangle which alterations observed in humans with OUD are driven by acute drug exposure and which alterations are consequences of chronic exposure.

## Introduction

Opioid use disorder (OUD) has emerged as a severe, ongoing public health emergency, with rapidly increasing prevalence rates driven by prescription opioids and recently, a surge in availability of synthetic opioids, such as fentanyl (1). The rate of overdose deaths due to any opioids has seen a parallel increase over the same time span reaching more than 80,000 overdose deaths per year (2). Current treatment strategies have proven inadequate to produce long-term abstinence for most users. Indeed, across different treatment strategies, most users relapse after initial sobriety, underscoring the lasting effects of chronic opioid exposure (3–5). This relapse propensity emphasizes the need to understand not only the mechanisms of drug seeking and taking, but also what occurs in the brain during acute and protracted withdrawal. Disentangling withdrawal mechanisms may allow for the development of novel therapies to reduce the negative consequences of withdrawal and promote long term abstinence.

Drug-induced neuroplasticity is thought to involve molecular adaptations to mesolimbic reward regions such as the nucleus accumbens (NAc) and higher-order executive regions such as the dorsolateral prefrontal cortex (DLPFC) (6–8). Chronic opioid use leads to a decrease in drug response. For instance, activation of opioid receptors leads to molecular events that promote desensitization and the development of tolerance, including a cascade of receptor adaptations involving phosphorylation, internalization, and a reduction in signal transduction which are the (9). Opioid exposure also produces an early epigenetic response. The promoter for the mu opioid receptor1 gene, *OPRM1*, is hypermethylated in response to short-term therapeutic use (10). Long-term opioid exposure leads to profound alterations in DNA methylation and gene expression (11), microRNA-mRNA network dysregulation (12), DNA damage (13), and dysregulation of angiogenic and cytokine gene networks (14). These epigenetic changes result in opioid effects that persist long after the drug has been metabolized. However, our knowledge of how these changes evolve from intoxication through withdrawal and into long-term abstinence in human disease is incomplete.

Previously, we found enrichment of differentially expressed (DE) transcripts involving neuroinflammation and extracellular matrix (ECM) remodeling in the DLPFC and NAc of individual with OUD. Using cell type deconvolution and weighted gene connectivity analysis (WGCNA), we identified an accompanying upregulation of microglia markers and increased connectivity of neuroinflammatory and ECM signaling (15). However, all the subjects in that study had opiates on board at time of death and included individuals who died of overdose. Similarly, preclinical models of molecular mechanisms are typically produced using tissue taken from rodents with drugs on board at time of sacrifice. Thus, it is difficult to disentangle whether transcriptional changes are a product of acute or chronic factors, and few studies focus on molecular changes in the withdrawal or abstinence periods.

Here, we used a translational approach to compare molecular alterations in the NAc between humans with OUD and two rodent models across distinct stages of opioid use and withdrawal (acute and prolonged) using differential gene expression and network-based approaches. We found that the molecular signature in the NAc of females with OUD mirrored effects seen in the NAc of female mice at all stages of exposure (while self-administering, during acute withdrawal, during prolonged withdrawal). We found that men with OUD showed strong overlap in expression profile with rats in only acute withdrawal. This study provides fundamental insight into the converging molecular pathways altered by opioids across species. Further, this work helps to disentangle which alterations observed in humans with OUD are driven by acute vs. chronic drug effects and which alterations may persist long after drugs are no longer in the system.

## Materials and Methods

### Transcriptomics datasets

We previously published the human OUD data used in the current cross-species comparisons (GEO Accession: GSE174409, ID: 200174409) (15). Details of mouse and rat opioid exposure paradigms are included in Supplementary materials.

### Differential Expression Analysis

Differential expression was assessed using limma (16). Comparisons were controls vs Intoxicated, controls vs. Withdrawal, and controls vs. Abstinence. Transcripts with p < 0.01 and log2 fold change (FC) > ± 0.26 were considered differentially expressed. We also utilized rank–rank hypergeometric overlap (RRHO) to assess the overlap of differential expression patterns between humans and rodent models at each phase of opioid exposure using RRHO2 (17, 18). RRHO is a threshold-free method that identifies overlap between two ranked lists of differentially expression genes, where the ranking is based on the −log10(p value) multiplied by the effect size direction.

### Identification of Co-expression Networks

We performed a weighted gene co-expression network analysis (WGCNA) to identify gene modules (19, 20). Module differential connectivity (MDC) was used to quantify differences in co-expression within modules between OUD and unaffected comparison subjects. To quantify module connectivity between each set of control-treatment groups, subsets of adjacent matrices were generated for treatment and control separately, and then directly compared using the ratio of summed lower triangular values. To assess this MDC metric statistically, we performed a permutation analysis that randomized values across the lower triangular matrix as well as the genes within each module. The output p-value for the MDC is computed by comparing the observed MDC to the corresponding MDC from the null distribution, which aids in determining whether a module significantly gained or lost connectivity – MDC > 1 indicates gain of connectivity, while MDC < 1 indicates loss of connectivity. We note that the data was permuted 1000 times in this analysis and that we considered two shuffling schemes to compute p values in this analysis: (1) shuffled samples—adjacent matrix with non-random nodes but random connections; (2) shuffled genes— adjacent matrix with random nodes but non-random connections. Computed p-values were converted to q-values following the Benjamini-Hochberg procedure, with q < 0.05 considered statistically significant. Fisher’s exact test determined whether DE transcripts were enriched within WGCNA modules. ARACNe was used to identify hub and OUD-specific hub genes for network analysis and Cytoscape was used to visualize networks. Pathway overrepresentation categories for each module was assessed using Metascape, with the 5000 WGNCA-analyzed genes as background.

## Results

**Figure 1:**
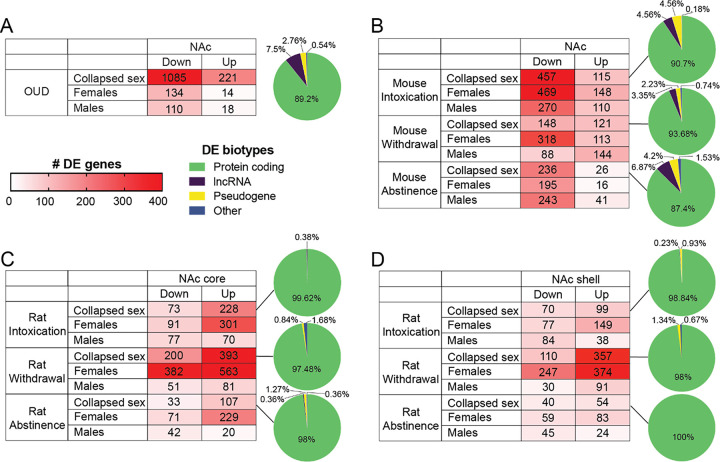
Transcriptomic changes in the NAc from human subjects with OUD and two rodent models of opioid-dependence. (A) Down and up-regulated transcripts in Human subjects with OUD with both sexes combined (top row) and then for each sex individually. Increasing number of DE genes (corrected p<0.01 and log2fold change ≤ −0.26 or ≥ 0.26) indicated by saturation of color. Pie chart shows biotypes of DE transcripts in the NAc. Protein coding genes represent the majority of transcripts followed by lncRNAs. (B) Down and up-regulated transcripts in mouse across three stages opioid exposure. (C) Down and up-regulated transcripts in rat NAc core across three stages of opioid exposure. (D) Down and up-regulated transcripts in rat NAc shell across three stages of opioid exposure. DE, differentially expressed; lncRNA, long noncoding RNA, NAc, nucleus accumbens; OUD, opioid use disorder.

Because corticostriatal circuit dysfunction contributes to the impairments that are a hallmark of substance abuse (21), we determined the impact of OUD on transcriptional differences in the human NAc. In line with our previous work, we found that many more NAc DE transcripts were downregulated than upregulated in OUD when collapsing all human subjects across biologic sex (Fig. 1A; 1085 down vs. 221 up). However, sex differences have been described across several domains in OUD including comorbidity with psychiatric symptoms (22), cue-induced craving/neural activity (23), and DNA methylation of OUD-related genes (24). To identify potential sex-specific transcriptional signatures of OUD, we examined the sexes separately. When disentangling the cohorts by sex, the pattern of downregulation was preserved. More transcripts were downregulated than upregulated in females (134 down vs. 14 up) and males (110 down vs. 18 up) (Fig. 1A). Most DE transcripts were protein coding (89.2%) with a much smaller fraction representing long non-coding RNAs (lncRNAs; 7.5%) and pseudogenes (3.3%).

We then investigated the effect of opioid administration in two rodent models (mice and rats) at three stages of opioid use and withdrawal. For the mouse study, we examined DE in three groups compared to naïve controls: 1) chronic self-administration and intoxicated at time of sacrifice (Intoxication); 2) chronic self-administration and in withdrawal for 24 hours (Withdrawal); 3) chronic self-administration and abstinent for two weeks (Abstinence). In the mouse NAc, opioid exposure was associated with more down than upregulated genes across all three opioid exposed groups (Fig. 1B). When separating by sex, both sexes recapitulated the pattern except for Withdrawal males, where the pattern was reversed (88 down vs 144 up). The majority of DE transcripts were protein coding genes followed by lncRNAs across the three stages of opioid exposure.

In our rat model (25), we examined the NAc core and shell separately. We examined DE in three groups compared to naïve controls: 1) chronic self-administration and intoxicated at time of sacrifice (Intoxication); 2) chronic self-administrations and in withdrawal for 24 hours (Withdrawal); 3) chronic self-administration and abstinent for 4–5 weeks (Abstinence). Unlike humans or mice who died with opioids on board (i.e., intoxicated), more DE transcripts were upregulated than downregulated in the NAc core of rats across the three opioid exposed groups (Fig. 1C). When separating by sex, the pattern of upregulation remained in females across the three stages of exposure. However, the ratio between DE transcripts was more balanced in males which could be a product of the relatively small number of DE transcripts in the NAc core of male rats. Similarly, in the rat NAc shell, there were more upregulated DE transcripts than downregulated across the three groups (Fig. 1D). However, when disentangling groups by sex, this pattern was reversed in Intoxicated and Abstinent male rats (Fig. 1D). For both rat NAc core and shell, greater than 97% of DE transcripts were protein coding across all three stages of opioid administration.

**Figure 2:**
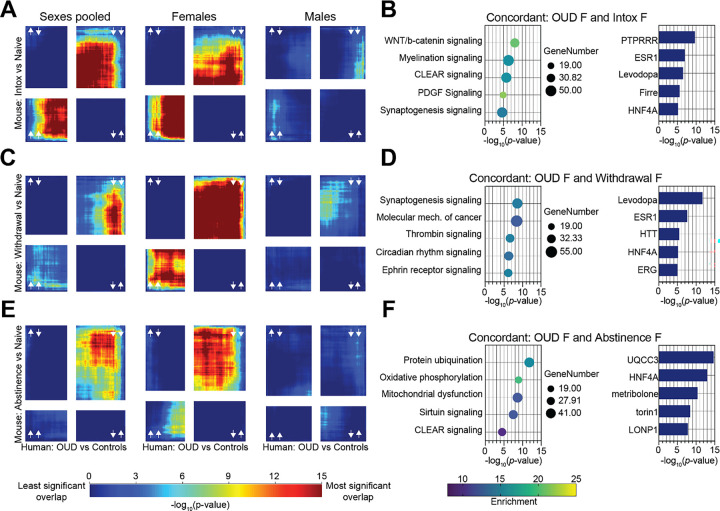
Transcriptional changes in NAc from female human OUD subjects overlap with mouse self-administration models. RRHO2 analysis was used to assess threshold-free overlap in gene expression between humans in our OUD cohort and mice that self-administered opioids. (A) RRHO plots showing concordant gene expression between human OUD subjects pooled by sex (left column), human female OUD (center column), and human male OUD (right column) and mice intoxicated ATOD. Increasing color warmth indicates increasing −log10 p-value. (B) Pathway enrichment analysis of transcripts concordant between OUD females and female mice intoxicated ATOD (left). Circle size corresponds to gene number and increasing color warmth indicates enrichment of −log10 p-value. Bar graph displays potential identified upstream regulators with −log10 p-value on the x axis (right). (C) RRHO plots showing concordant gene expression between human OUD subjects pooled by sex (left column), human female OUD (center column), and human male OUD (right column) and mice abstinent ATOD. (D) Pathway enrichment analysis of transcripts concordant between OUD females and female mice in acute withdrawal ATOD (left). Bar graph displays potential identified upstream regulators (right). (E) RRHO plots showing concordant gene expression between human OUD subjects pooled by sex (left column), human female OUD (center column), and human male OUD (right column) and mice in acute withdrawal ATOD. (F) Pathway enrichment analysis of transcripts concordant between OUD females and female mice abstinent ATOD (left). Bar graph displays potential identified upstream regulators (right). ATOD, at time of death; NAc, nucleus accumbens; OUD, opioid use disorder; RRHO, rank-rank hypergeometric overlap.

Next, we interrogated whether the pattern of gene expression in human OUD mapped onto the signature from our mouse opioid exposure groups using threshold-free RRHO analysis. These analyses have the potential to identify which transcriptional changes in human OUD are a product of the acute effects of opioid intoxication versus the long-term effects of chronic use. When the sexes in our human cohorts were pooled, there was substantial overlap in both upregulated and downregulated transcripts between humans and Intoxicated mice. However, when splitting by sex, OUD females but not males showed strong concordance with sex-matched Intoxicated mice (Fig. 2A). The same pattern was true for mice in Withdrawal (Fig. 2C) and Abstinence (Fig. 2E). Pathway enrichment was performed on shared transcripts between OUD females and female mice across the three mouse groups (Fig. 2B, D, F). Top shared pathways included Wnt/β-catenin, myelination, CLEAR (Coordinated Lysosomal Expression and Regulation), Platelet-derived growth factor (PDGF)-mediated signaling, and synaptogenesis signaling. We identified genes which act as potential upstream regulators in our mouse groups. In Intoxicated and Withdrawal mice, we identified levodopa (L-DOPA), the central precursor in the synthesis of the catecholamine neurotransmitters dopamine, norepinephrine, and epinephrine as an upstream pathway regulator (Fig. 2B, D). We also identified the gene for Estrogen receptor alpha (ERα), *ESR1*, as a potential upstream regulator in Intoxicated and Withdrawal mice (Fig. 2B, D). When activated by its ligands, ERα acts a transcription factor with roles across the organism (26). The *HNF4A* gene which also encodes a transcription factor, emerged as an upstream regulator across all three opioid exposed groups.

**Figure 3:**
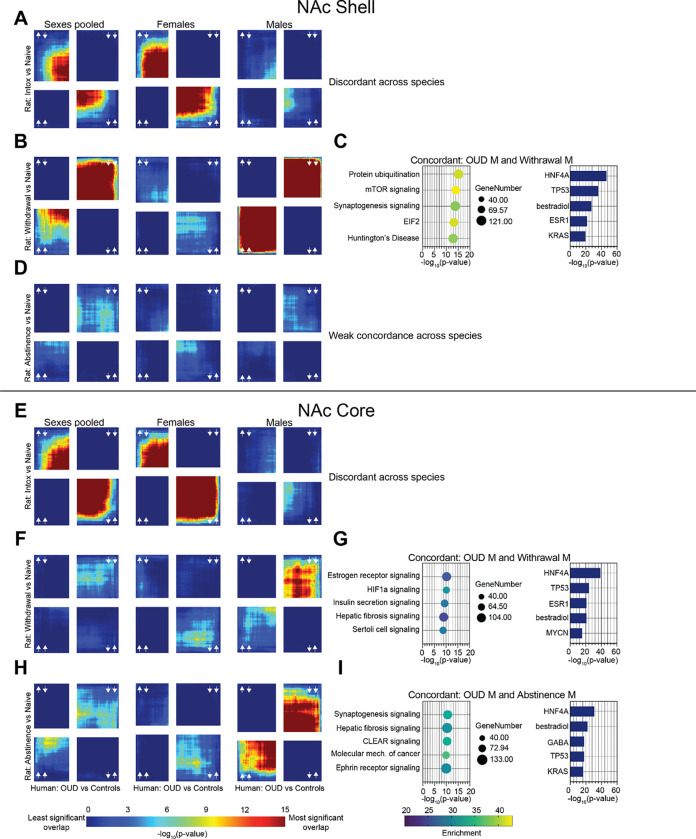
Transcriptional changes in NAc from male human OUD overlap with withdrawal and abstinence rat self-administration models. (A) RRHO plots showing concordant gene expression between human OUD subjects pooled by sex (left column), human female OUD (center column), and human male OUD (right column) and NAc shell of rats intoxicated ATOD. (B) RRHO plots showing concordant gene expression between human OUD subjects pooled by sex (left column), human female OUD (center column), and human male OUD (right column) and NAc shell of rats in acute withdrawal ATOD. (C) Pathway enrichment analysis of transcripts concordant between OUD males and NAc shell of male rats in acute withdrawal ATOD (left). Bar graph displays potential identified upstream regulators (right). (D) RRHO plots showing concordant gene expression between human OUD subjects pooled by sex (left column), human female OUD (center column), and human male OUD (right column) and NAc shell of rats abstinent ATOD. (E) RRHO plots showing concordant gene expression between human OUD subjects pooled by sex (left column), human female OUD (center column), and human male OUD (right column) and NAc core of rats intoxicated ATOD. (F) RRHO plots showing concordant gene expression between human OUD subjects pooled by sex (left column), human female OUD (center column), and human male OUD (right column) and NAc core of rats in acute withdrawal ATOD. (G) Pathway enrichment analysis of transcripts concordant between OUD males and NAc core of male rats in acute withdrawal ATOD (left). Bar graph displays potential identified upstream regulators (right). (H) RRHO plots showing concordant gene expression between human OUD subjects pooled by sex (left column), human female OUD (center column), and human male OUD (right column) and NAc core of rats abstinent ATOD. (I) Pathway enrichment analysis of transcripts concordant between OUD males and NAc core of male rats abstinent ATOD (left). Bar graph displays potential identified upstream regulators (right). ATOD, at time of death; NAc, nucleus accumbens; OUD, opioid use disorder; RRHO, rank-rank hypergeometric overlap.

We then turned to opioid exposed rats and used the same approach as used for mice to interrogate the relationship of gene expression to human OUD and probe how that relationship differs from that of opioid exposed mice. Unlike in humans and mice, we looked at the NAc shell and core separately in our rat groups. In the NAc shell, the pattern of gene expression was strongly discordant between humans and Intoxicated rats when the sexes in our human cohorts were pooled. When the sexes were separated, gene expression was still discordant but more strongly in women than in men (Fig. 3A). Gene expression was strongly concordant between humans and Withdrawal rats. However, this seems to be driven by OUD men given that when the sexes were examined separately, only OUD men showed concordance with male Withdrawal rats. OUD women were weakly discordant with female Withdrawal rats (Fig. 3B). The same overall patterns held consistent between humans and abstinence rats. Gene expression was weakly concordant between all humans and Withdrawal rats but when separated by sex, males were weakly concordant, and females were weakly discordant (Fig. 3D). Pathway enrichment was performed on shared transcripts between OUD males and male Withdrawal rats (Fig. 3C). Top pathways included protein ubiquitination and mTOR signaling. We identified upstream regulators for the Withdrawal group. Surprisingly, two genes, *ESR1* and *HNF4A*, which we identified as upstream regulators in our concordant mouse pathways, popped up here as well.

In the NAc core, identical to the NAc shell, the pattern of gene expression was strongly discordant between humans and Intoxicated rats when the sexes in our human cohorts were pooled. However, when the sexes were separated, gene expression was strongly discordant in females and weakly discordant in males (Fig. 3E). Also, like the NAc shell, gene expression was strongly concordant in OUD males and male Withdrawal rats, weakly discordant between OUD females and female Withdrawal rats, and weakly concordant between all human OUD subjects and Withdrawal rats (Fig. 3F). The same was true between human OUD subjects and Abstinence rats (Fig. 3H). Pathway enrichment was performed on shared transcripts between OUD males and male Withdrawal and Abstinence rats (Fig. 3G, I). While there were no shared pathways between Withdrawal and Abstinence groups, two of the top pathways in the Abstinence comparison, synaptogenesis and CLEAR signaling, emerged in the Withdrawal comparison in the rat NAc core, and across the three comparisons in the mouse groups, suggesting the importance of both pathways in the development of tolerance across species (Fig. 3I). We identified upstream regulators for the Withdrawal and Abstinence groups. *HNF4A*, emerged again across both groups which indicates the importance of this gene’s regulatory role across sex and species. We identified *ESR1* as an upstream regulator in the NAc core of Withdrawal rats but not Abstinence rats (Fig. 3G, I). Upstream regulators were highly consistent across the NAc shell and core. *TP53*, a tumor suppressing gene which acts as a transcriptional activator, and bestradiol (estradiol), a ligand for the estrogen receptor, were identified as upstream regulators across both regions (Fig. 3C, G, I).

**Figure 4:**
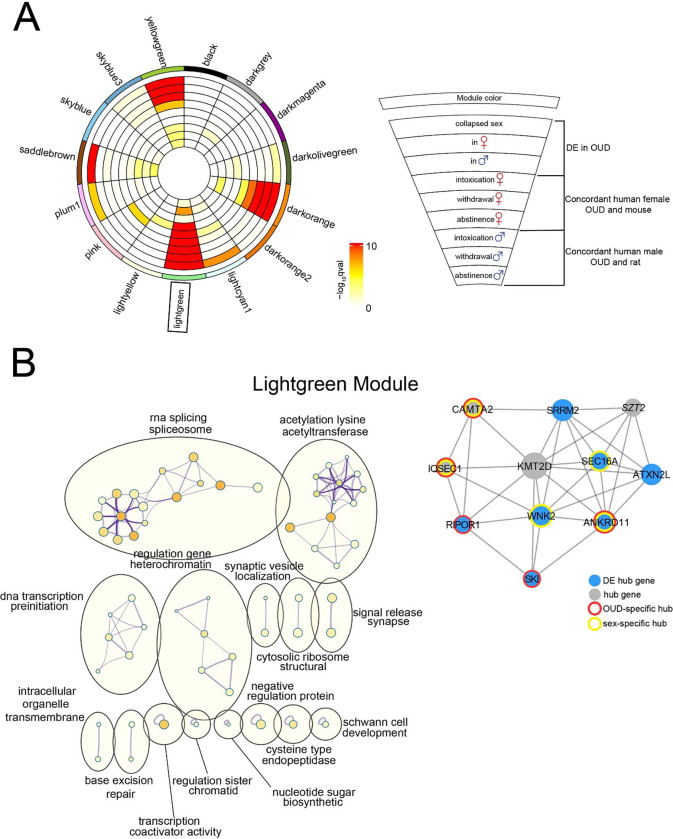
OUD-associated gene networks in the NAc. (A) Co-expression modules and network structure of transcription in the NAc was generated using WGCNA. Circos plot identified by module names and colors. Enrichment for transcripts DE in OUD, concordant human female and mouse, and concordant human male and rat are indicated by semi-circle colors within each module, with increasing warm colors indicating increasing −log_10_ p-value. (B) Left: Network visualization generated using Cytoscape with categorical labels for multiple clusters. Right: Hub gene coexpression networks of the NAc in the lightgreen module. Node size indicates the degree of connectivity for that gene. Blue nodes indicate DE hub genes. Gray nodes indicate hub genes. Red halos indicate OUD-specific hub genes. Yellow halos indicate sex-specific hub genes. DE, differentially expressed; NAc, nucleus accumbens; OUD, opioid use disorder.

To investigate gene correlations in human OUD and our two rodent models, we interrogated our gene networks using WGCNA. We first built the WGCNA modules using the human OUD data and then looked for enrichment in OUD-associated DE transcripts as well as cross-species concordance patterns within each module. There was strong enrichment of DE transcripts across all human OUD subject groups in the darkorange, yellowgreen (Supplementary Fig. 4), and lightgreen modules (Fig. 4A). In addition to being enriched for DE transcripts in all our human OUD subject groups, the lightgreen module showed a high prevalence of DE transcripts that were concordant between human female OUD and mouse as well as human male OUD and rat (Fig. 4A). Because this module showed the strongest enrichment for DE transcripts, we focused our attention there. We visualized the pathway network associated with the lightgreen module and identified clusters with significantly enriched terms based on pathways (Fig. 4B). The two largest clusters were categorized as RNA splicing/spliceosome and acetylation lysine/acetyltransferase. Other network clusters included ‘DNA transcription preinitiation’, ‘regulation gene heterochromatin’, ‘cytosolic ribosome structural’, and ‘transcription coactivator activity’. Together, this network analysis underscores the profound effect opioid exposure has on the regulatory mechanisms of DNA transcription from epigenetic modifiers through post-transcriptional regulation of RNA splicing. Other notable network clusters involved synaptic activity: ‘synaptic vesicle localization’ and ‘signal release synapse.’

To distinguish potential regulators of this co-expression network, we identified highly connected hub genes within the lightgreen module that were predicted to drive the expression of other genes within the module (Fig. 4B). Of the 11 identified hub genes, 5 were specific to OUD: *ANKRD11, CAMTA2*, *IQSEC1*, *RIPOR1*, and *SKI*. In addition, we also identified sex-specific hub genes as part of our network analysis. Three of those genes overlapped OUD hub genes: *CAMTA2*, *IQSEC1*, and *ANKRD11* while the other two, *SEC16A* and *WNK2*, were uniquely sex specific. The central hub gene in our network is *KMT2D*, a histone methyltransferase with a direct relationship with the estrogen receptor (27). This is particularly interesting given that one of the largest clusters in the lightgreen module is acetylation lysine/acetyltransferase, further suggesting opioids lead to heterochromatin modifications (15, 28).

**Figure 5:**
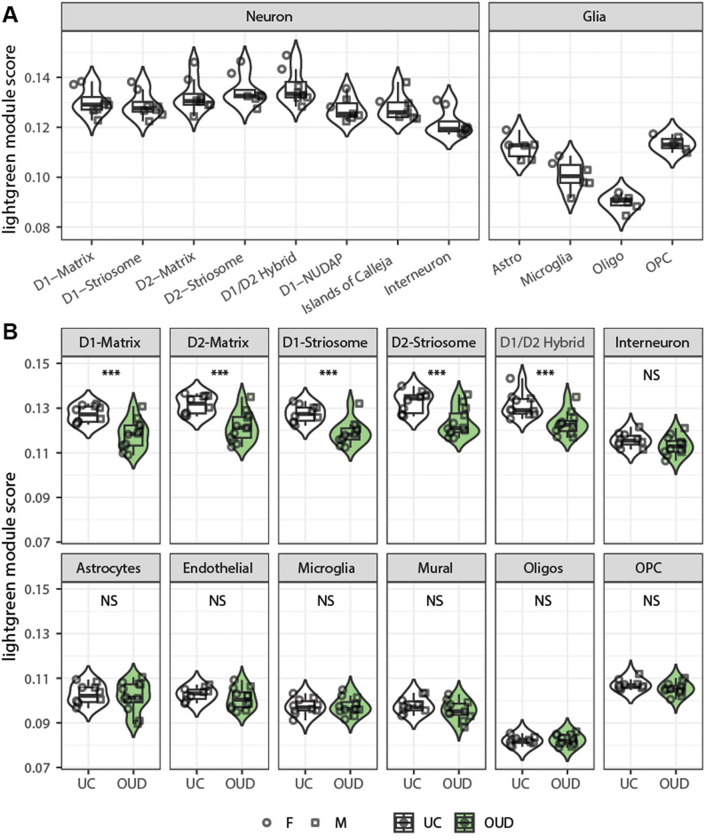
Cell type-enrichments identifies opioid-related gene network alterations in striatal medium spiny neurons across mouse, rat, and human. (A) Area under curve for cell types (AUCell) of the lightgreen module identified by WGCNA derived from human striatum with y-axis representing the relative expression of module gene score (29). Each major neuronal (left) and glial (right) cell subtypes are represented. Higher module scores in neurons reflect preferential enrichment of genes in the lightgreen module within neurons of human striatum relative to glial cells. (B) AUCell of the lightgreen module genes are decreased in expression primarily in medium spiny neuron subtypes (matrix and striosome neurons that expression dopamine receptor 1 or 2) in individuals with OUD. Y-axis represents the relative expression of module gene score. D1 or D2, dopamine receptor 1 or 2 expressing neurons; neurochemically unique domains in the accumbens and putamen (NUDAP); Astro, astrocytes; Oligos, oligodendrocytes; OPC, oligodendrocyte precursor cells; F or M, females or males, respectively; UC, unaffected control individuals; OUD, individuals with opioid use disorder (OUD); NS, non-significant between UC and OUD cohorts; ***, significantly different between UC and OUD cohorts at p<0.001).

The lightgreen module represents a potential interacting, correlative gene network that is integrated across mouse, rat, and human. To further deconvolve the cell types of represented by the genes in the lightgreen module, we assessed whether module genes were enriched for specific cell types in human striatum (29, 30). Overall, the lightgreen module was enriched for genes in neuron subtypes compared to glial cells (Fig. 5A), suggesting the module is composed mainly of genes active in neurons across species in response to opioids. In OUD, module genes were significantly reduced in enrichment within medium spiny neurons relative to interneurons and glial cells (Fig. 5B). Enrichment changes were independent of sex (Fig. 5B), occurring in both females and males with OUD. Thus, module genes were decreased in expression in medium spiny neurons in OUD, reflecting similar genes and consistent correlative expression across the gene network in medium spiny neurons across rodent models of opioids and individuals with OUD.

## Discussion

Here, we demonstrate sex specificity in concordance of the molecular signature of OUD in the human NAc and the molecular signature of opioid-exposure in preclinical models. OUD females mirrored effects seen in the NAc of female mice at all stages of opioid exposure, while OUD males showed strong overlap in expression profile with male rats in acute Withdrawal (NAc shell, core) and Abstinence (NAc core).

Two top shared pathways, synaptogenesis and CLEAR signaling, were consistently identified in concordant genes across species and opioid exposure, suggesting opioid dependence is closely linked with synaptic alterations and regulation of lysosomal degradation. WGCNA identified co-expression networks involved in post-transcriptional modification of RNA and epigenetic modification of chromatin state, underscoring the effect of opioid exposure on transcriptional regulation.

While epidemiologic studies have consistently demonstrated a higher prevalence of illicit drug use in males, evidence suggests that drug use in women transitions more quickly from casual, recreational use to dependence (31–33). Specific to opioids, females report higher levels of baseline and cue-induced craving (23, 34, 35), more physical withdrawal symptoms, greater functional impairment, and higher levels of comorbid psychological suffering (36–38). Preclinical studies show that females acquire opioid self-administration more quickly, self-administer more, reach higher breakpoints during fixed ratio, and respond more during extinction and cue-induced reinstatement (39–45). Our work provides added nuance to understanding sex differences in OUD. When comparing human subjects to our rodent models of disease, OUD females were concordant with female mice, while OUD males were concordant with male rats. While mechanisms of opioid dependence appear to be unique to sex and species, several patterns emerged with regards to top shared pathways in concordant genes and their upstream regulators. Estrogen receptor signaling was the strongest pathway finding in the rat NAc core, and both ESR1, the gene for estrogen receptor alpha (ERα), and a ligand for this receptor, bestradiol (trade name for the endogenous steroid hormone estradiol) were identified as upstream regulators across sex and species. ERα is the predominant estrogen receptor subtype and estradiol is the primary circulating estrogen hormone. When bound, the receptor undergoes a conformational change, homodimerizes, and together estradiol and ERα form a complex which binds the promoter region of target genes to regulate their expression (26, 46). Evidence suggests that gonadal hormones and estradiol specifically play a role in guiding behavior around substance use (47–51). Female rats take a similar number of heroin infusions during estrus, metestrus, and diestrus, but self-administer significantly less during proestrus when estradiol is at its peak (43). Ovariectomy (OVX) removes the primary source for estrogens and, in mice, increases morphine conditioned place preference (CPP) at a 10 mg/kg dose, which is reversed by estradiol replacement (52). These findings suggest that estradiol leads to an overall reduction in drug seeking/reward. However, the data are complex. At smaller doses of morphine (2,5 mg/kg), OVX in female mice decreases CPP and estradiol replacement increases CPP (52). Following estradiol replacement in OVX rats, animals acquired opioid SA more quickly, self-administered higher levels of opioids, and showed an enhanced sensitivity to opioid-related cues compared to vehicle-treated OVX control (53, 54), which is in keeping with estradiol’s effect in studies using psychostimulants and alcohol (55–60). However, other studies have found no effect of OVX in rat opioid self-administration models (61, 62). Together this suggests that gonadal hormones play some role in the rewarding effects of drugs of abuse, but the relationship to opioids needs further study. One potential mechanism is through regulation of synaptic density. Estradiol and the primary male gonadal hormone testosterone can modulate spine plasticity in both NAc core and shell (32, 63–66). Notably, synaptogenesis signaling emerged as a consistent pathway finding across sex, species, and opioid exposure group in our analyses. It is interesting to speculate how gonadal hormones may modulate the rewarding effect of drugs of abuse through altering baseline spine density in reward-related cortical regions.

In keeping with the idea that the long-lasting effects of opioid exposure are mediated through epigenetic modification, the central hub gene in our network is *KMT2D*, a histone methyltransferase with a direct relationship with ERα. As discussed above, activated ER forms a complex with other transcription factors and binds chromatin at specific sequences. KMT2D actively modulates chromatin, creating a more open chromatin state so the activated ER complex can be recruited to ER response elements in the genome and promote ER-dependent transcription (27, 67). This is interesting because it is a potential link between epigenetic regulation on one hand, and sex specificity on the other. Sex differences have previously been described in the epigenetic regulation of opioid-related genes. In a retrospective study of 250 outpatients who experience chronic non-cancer pain, there were sex differences in methylation of the promoter region the μ-opioid receptor 1 gene *OPRM1* which was significantly correlated to lower rates of OUD in females (24). Unpacking the mechanisms by which opioid exposure modulates the ability of transcriptional regulators to interact with chromatin is integral for understanding how environment and experience shape gene expression in the context of addiction.

Regardless of sex or species, HNF4A (hepatic nuclear factor 4, alpha) was identified as an upstream regulator across all concordant pathways. HNF-4α is a nuclear transcription factor that binds DNA as a homodimer and plays a role in the development of several major organ systems including the liver, kidneys, and intestines (68–71). Converging lines of evidence implicate HNF4A in the development of several kinds of cancer (72–74). Specifically, HNF4A interacts with the Wnt-β catenin pathway to guide cell adhesion, cell proliferation, apoptosis, and tumorigenesis (75–77). Interestingly, Wnt-β catenin was the strongest pathway finding in our OUD female and mouse female Intoxication comparison, and molecular mechanisms of cancer emerged in several of our concordance findings. It is currently unclear what this means for the development of dependence, but the consistency of this finding suggests that HNF4a should be a focus of future study in subsequent OUD work.

We identified highly connected hub genes within the lightgreen module that were specific to OUD: *ANKRD11, CAMTA2, IQSEC1, RIPOR1*, and *SKI*. Previous evidence links the activity and regulation of *CAMTA2, IQSEC1*, and *RIPOR1* to OUD. CAMTA2 is a member of the ubiquitous Ca^2+^/calmodulin-dependent protein kinase family (CaMKs), which are crucial players in producing the synaptic changes that underlie the formation of memory (78–80). Though CAMTA2 has not been directly linked to opioid addiction, this protein family generally, and one member, CAMKII specifically, have been both linked to the development of opioid tolerance and dependence (81–85). CAMKII activity is increased by morphine treatment and inhibiting CaMKII in rat hippocampus attenuates morphine tolerance and dependence (86, 87). IQSEC1 was identified as a potential regulator for receptor endocytosis by KEGG analysis of putative targets for responsive NAc miRNAs after morphine self-administration (88), which has direct relevance to μ-opioid receptor desensitization which is a molecular correlate of the development of dependence. Last, RIPOR1 is a member of the Rho family of GTPases which regulate cellular processes such as cytoskeleton remodeling and synapse maintenance (89–91). Drugs of abuse, including morphine and heroin, alter the activity of these proteins (92). In addition, we compared the genes from our lightgreen module to genes linked to OUD by existing GWAS work. Two stood out as particularly interesting, *GABRG2* and *FURIN*. *GABRG2* encodes a gamma-aminobutyric acid (GABA) receptor subunit. Not only has *GABRG2* been linked to both alcohol use disorder and OUD (93, 94), but we previously demonstrated a microglia-specific upregulation of *GABRG2* in OUD using single nuclei transcriptomics in human OUD (30). FURIN is a proteolytic enzyme, and notably has a single variant association with OUD as identified through GWAS (95–98).

Our data provide crucial nuance to understanding sex-specific mechanisms of OUD in human subjects. Together, our findings demonstrate how the relationship between opioid use, gene expression and its regulation through epigenetic modifications is informed by input from gonadal hormones with consequences for synaptic modeling in corticostriatal circuitry Further, these results suggest the species-specific pattern of concordance potentially determines the efficacy of choosing one model species over another when designing preclinical studies to investigate mechanisms with relevance to human OUD. This work deepens our understanding of sex specificity in OUD and opens the door for future research into putative therapies.
